# Dr. Sherman Voorhees

**Published:** 1915-06

**Authors:** 


### OBITUARY

**Readers are requested to report promptly the death of all physicians in Western New York, or former residents of this region, or graduates of any medical school in Western New York, and to notify the families of the deceased of our desire to publish adequate obituary notices.**

**Dr. Sherman Voorhees,** P. & S. Baltimore, 1893, of Elmira, died at the home of his sister, Dr. Belle V. Aldridge, 32 Clarkson Ave., Brooklyn, at midnight, May 1-2. July 5, 1914, he and his wife plunged down a 100 foot embankment in their auto. His wife was killed and Dr. Voorhees suffered a fracture of the pelvis and other injuries. Death was due to the remote effects of the physical and mental shock and to some lesion, probably fracture of the base of the skull, involving the vital centers. He was well known as an ophthalmologist and otologist.

